# Recent Progress on Circular RNA Research in Acute Myeloid Leukemia

**DOI:** 10.3389/fonc.2019.01108

**Published:** 2019-11-06

**Authors:** Muhammad Jamal, Tianbao Song, Bei Chen, Muhammad Faisal, Zixi Hong, Tian Xie, Yingjie Wu, Shan Pan, Qian Yin, Liang Shao, Qiuping Zhang

**Affiliations:** ^1^Department of Immunology, School of Basic Medical Science, Wuhan University, Wuhan, China; ^2^Institute of Pathology, Hannover Medical School, Hanover, Germany; ^3^Department of Hematology, Zhongnan Hospital of Wuhan University, Wuhan, China; ^4^Hubei Provincial Key Laboratory of Developmentally Originated Disease, Wuhan University, Wuhan, China

**Keywords:** acute myeloid leukemia (AML), circular RNA (circRNA), biomarker, apoptosis, cell proliferation, drug resistance, targeted therapy

## Abstract

Acute myeloid leukemia (AML) is a myeloid malignancy characterized by the proliferation of abnormal and immature myeloid blasts in the bone marrow. Circular RNA (circRNA) is a novel class of long non-coding RNA with a stable circular conformation that regulates various biological processes. The aberrant expression of circRNA and its impact on AML progression has been reported by a number of studies. Despite recent advances in circRNA research, our understanding of the leukemogenic mechanism of circRNA remains very limited, and translating the current circRNA-related research into clinical practice is challenging. This review provides an update on the functional roles of and research progress on circRNAs in AML with an emphasis on mechanistic insights. The challenges and opportunities associated with circRNA-based diagonostic and therapeutic development in AML are also outlined.

## Introduction

Acute myeloid leukemia (AML) is the most common leukemia in adults, presenting great biological and clinical heterogeneity (1). In 2018, there were 19,520 new cases of AML in the United States (2), and, based on survey data, there were 14,100 reported cases in China in 2015 (3). Various genetic and epigenetic aberrations arrest hematopoietic cell differentiation and maturation events, leading to the accumulation of abnormal and immature hematopoietic progenitor cells in the bone marrow and peripheral blood. These abnormalities are often associated with lethal infection, organ infiltration, and cytopenias (4, 5).

Investigations on the genetic and molecular mechanisms of cancer have recently shifted from protein-coding genes to non-coding transcripts (6). About 98% of the human genome represents non-coding DNA sequences, and the majority of the human transcriptome is classified as non-coding RNAs (ncRNAs) (7). Circular RNA (circRNA) belongs to the family of endogenous ncRNA and has a non-polyadenylated closed single-stranded and continuous loop structure due to a covalent phosphodiester bond between the 3′ and 5′ ends (8). The size of circRNA ranges from a few hundred to thousands of nucleotides (9). Global circRNA expression profiling has revealed that they are abundant transcripts of well-regulated back-spliced RNA and that their biogenesis is regulated by cis-elements and/or trans-factors. The back-spliced RNA is produced as a result of back-splicing that ligates a splice donor site with an acceptor site present upstream and downstream, respectively, on the mRNA transcript, resulting in a covalently closed circRNA as well as an alternatively spliced linear RNA with skipped exon(s) (1). The circular and non-polyadenylated structure makes the circular RNA more stable. circRNAs exhibit evolutionary conservation across the eukaryotes (10), and circRNA expression is tissue- and developmental stage-specific (11–13). Studies have shown that circRNAs regulate gene expression (14, 15) and are involved in regulating vital cellular events such as differentiation, proliferation, growth, signaling, and aging (11–13, 16, 17). Recently, aberrant expression of circRNAs has been implicated in the progression and pathogenesis of hematopoietic malignancies (18–20) and solid tumors (21–23). Numerous circRNAs with altered expression have been reported to be involved in leukemogenesis (24–26). miRNAs, short stretches of RNA (23 nt), are associated with various biological processes (2). circRNAs are also implicated in tumorigenesis, metastasis, and drug resistance (22). The most established mechanism of action of circRNAs is its “sponge” function via binding to miRNAs (14, 27), proteins (21, 28, 29), or DNA (10, 30). The most documented role of circRNAs in AML is modulating mRNA stability and translation by sequestering the mRNA transcript and protein (31, 32), although circRNA-RNA binding protein (RBP)/DNA interactions remain obscure and need further exploration.

This review compiles the roles of recently reported circRNAs that show altered expression in AML. Moreover, we also present the potential molecular mechanisms of certain circRNAs. Finally, we discuss the main challenges related to research on circRNA.

## Biogenesis and Functions of circRNA

The unique characteristic of circRNAs is associated with their production, which mostly occurs via the canonical and non-canonical splicing (3) of exons (4) and introns, non-coding antisense, 3′ and 5′ UTR or the intergenic region (4, 5). Non-canonical splicing is an unconventional mechanism of splicing characterized by the ligation of splice sites that are present at a distance from the currently annotated exons. The process aids in gene expression regulation and is important in evolution since it acts as a source of newly emerging transcripts (6).

**Figure 1:**
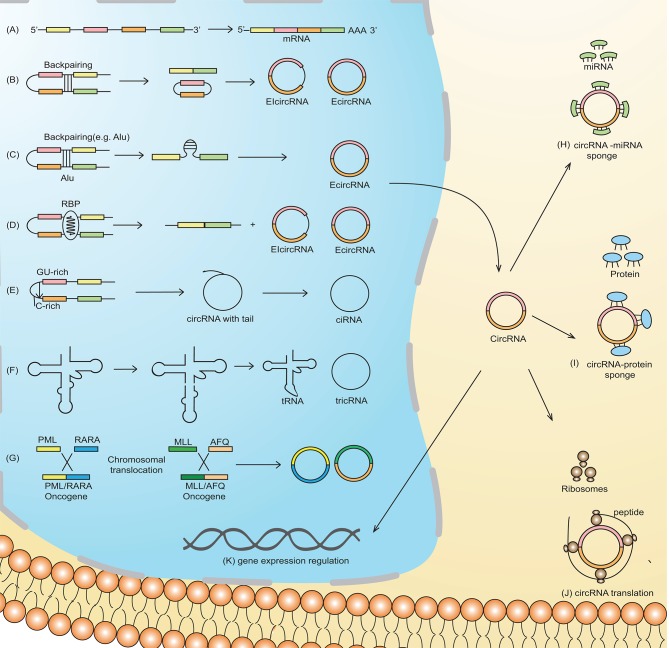
Schematic representation of circRNA biogenesis. **(A)** Canonical pre-mRNA producing a mature mRNA. **(B)** Lariat-driven circularization, also called exon-skipping, is a process in which the partial folding of pre-mRNA allows the covalent binding of the exonic 5′-splice donor site with the 3′-end acceptor site, thus resulting in the skipping of exons. **(C)** Intron-pairing-driven circularization is characterized by base-pairing between different repetitive elements such as Alu repeats across different introns. The intronic sequence is subsequently cleaved to produce exonic circRNA (ecircRNA). **(D)** RNA-binding proteins (RBP) working as *trans*-acting factors vitally regulate the production of circRNA. Quaking (QKI) and Muscleblind (MBL/MBNL1) proteins preferentially bind to a particular sequence motif on a linear RNA, thus bridging the two bracketing introns together and circularizing the linear RNA to generate circRNA. **(E)** A novel circRNA known as ciRNA is dependent on a 7-nt GU-rich motif and an 11-nt C-rich sequence near the 5′-splice site and branchpoint site. The lariat structure containing the excised intron and exons is trimmed by the spliceosome followed by the degradation of the 3′-end tail, ultimately generating ciRNA. **(F)** tRNA intronic circular RNAs (tricRNAs) are produced as a product of the splicing of tRNA independent of pre-mRNAs, which is catalyzed by an enzyme in a presence of conserved tRNA sequence motif. **(G)** Chromosomal translocation results in the fusion of two genes producing fusion circRNA (f-circRNA). Functions of circRNA: **(H)** circRNAs may act as miRNA sponges by competing for miRNA binding sites, diminishing the effect of miRNA-mediated regulatory activities. **(I)** cricRNA may acts as protein (RNA binding protein), which regulate gene expression and are thus involved in various biological processes **(J)** circRNAs can be translated to form functional proteins, e.g., ribosome-associated circRNAs (ribo-circRNAs) harbor start codon and evolutionarily conserved stop codon encoding at least in protein domain circRNAs. **(K) c**ircRNAs (e.g., EIciRNAs and ciRNAs) may interact with transcription complexes and enhance the expression of their parental genes. Moreover, some of the nuclear enriched circRNAs may bind with the genomic DNA, forming an RNA-DNA triplex that can regulate the DNA replication process.

Based on their origin of production and their determination by RNA-sequencing (RNA-seq), the circRNAs are classified into four types: exonic circRNA (ecircRNA) (4, 7), circular intronic RNA (ciRNA) (5), retained-intron or exon-intron circRNA (7, 8), and intergenic circRNA (4). Two models of circularization, illustrated in Figure 1, explain circRNA biogenesis via lariat-driven circularization (exon skipping) (Figure 1B) (9) or intron pairing-driven circularization (Figure 1C) (9). circRNA biogenesis is regulated by both *cis-* and *trans*-acting factors (33), with the latter also known as RNA binding proteins (RBP) (Figure 1D) (10). Some circRNAs, in which the introns are not trimmed and are retained in exons, are known as exon-intron circRNAs or EIciRNAs (Figure 1D) (8). Unique splicing mechanisms may also result in the generation of novel circRNAs known as ciRNA (intronic circRNA) (5) (Figure 1E) and tRNA intronic circular RNAs (tricRNAs) (Figure 1F) (11).

The most established function of circRNAs is through sponge formation with miRNAs and proteins (Figure 1H). In the case of circRNA-miRNA sponge formation, the sequestration of miRNA by the circRNA allows the binding of translational machinery to the specific mRNA, thus causing gene derepression. Increased expression of genes that, for example, are associated with migration, differentiation, and proliferation, may contribute to leukemogenesis (10). Direct RBP sponging (circRNA-RBP) (Figure 1I) or indirect circRNA-miRNA-RBP interactions by circRNAs can also induce leukemogenesis, because RBPs are also implicated in cell cycle progression and the biogenesis of circRNA, ultimately thereby affecting cell differentiation, proliferation, and apoptosis (12).

Although circRNAs contain an open reading frame (ORF), they generally lack the essential components necessary for translation, such as a poly(A) tail and a 7-methylguanosine cap. Nevertheless, mounting evidence suggests the translation ability of circRNAs (Figure 1J) (13). For example, an RNA modification motif, N6-methyladenosine (m6A), enriched in circRNAs, facilitates the translation of circRNA in human cells (14). Overall, the translational mechanisms of circRNAs are not well-established and need further investigation.

## circRNAs in AML

### Circular RNAs as Potential Diagnostic and Prognostic Biomarkers (Table 1)

**Figure 2:**
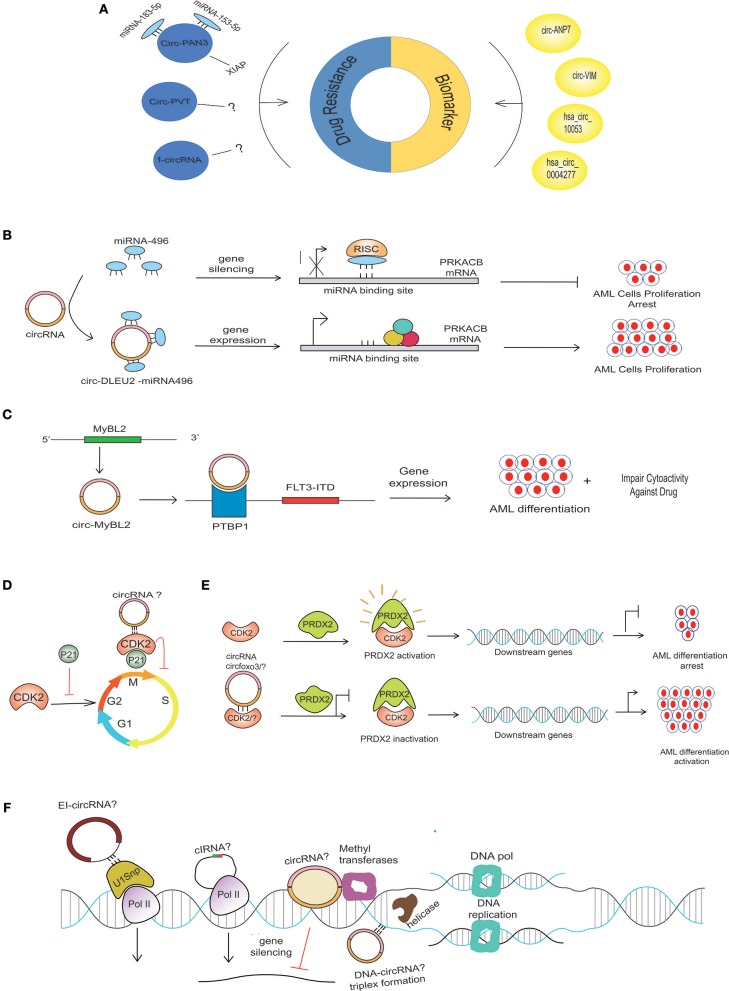
Possible mechanism of circRNA in AML pathogenesis. **(A)** Aberrant expression of various circRNAs detected in BM samples of AML patients can be implicated in the diagnosis and prognosis of AML. circRNAs use different mechanisms to contribute to drug resistance in AML. CircPAN3 binds with two different miRNAs (miRNA-183-5p and miRNA-153-5p) to increase the expression of XIAP, which is demonstrated to be a drug-resistance gene in AML. Several circRNAs generated from fused genes, known as fusion circRNA (f-circRNA), can also contribute to drug resistance in an unknown mechanism. CircPVT and f-circRNA (a product MLL/AF9) also potentiates drug resistance in an unknown mechanism. **(B)** miRNA-496 bind to their respective sites on mRNA, causing PRKACB repression, but circDLEU2-miRNA-496 sponge formation allows the binding of translational machinery to the mRNA, thus causing gene derepression. The increased expression of PRKACB is associated with the proliferation of AML cells. **(C)** CircMYBL2 produced from the MYBL2 gene positively regulates the expression of the FLT3-ITD mutant gene by recruiting PTBP1 to the mRNA of the FLT3-ITD gene. **(D)** CDK2, a cyclin-dependent kinase enzyme regulating the cell cycle, is negatively regulated by P21. Circ-Foxo3 binds to CDK2 inhibitor 21 (p21), forming circfoxo3-p21-CDK2 complexes, thus preventing cell cycle progression. **(E)** Interaction of PRDX2 with CDK2 causes PRDX2 activation that, in turn, regulates the downstream genes, thereby arresting AML cell differentiation; however, the depletion of CKD2 due to its possible binding with circRNA? or degradation could diminish its interaction with PRDX2, thus rendering PRDX2 to its inactivated stage. The inactivation of PRDX2 is associated with AML differentiation activation (36). **(F)** Possible circRNAs with various gene expression-regulating abilities in AML. CircRNAsX (e.g., EIciRNAs and ciRNAs) may interact with transcription complexes and enhance the expression of their parental genes; other circRNA (EcircRNA) may repress the gene expression. Certain circRNA, like the lncRNA, may interact with the DNA by forming a circRNA-DNA triplex to regulate DNA replication.

Various molecular-based biomarkers (e.g., cytogenetics, genetics, epigenetics, proteomics, and miRNA) have been reported in AML and have been comprehensively reviewed (15). circRNAs are emerging as potential biomarkers in the diagnosis and treatment of AML (Figure 2A) due to their stability against exonucleolytic degradation, tissue-specific expression patterns, and abundance in body fluids and exosomes. For example, microarray-based expression analysis of five bone marrow (BM) samples from AML patients revealed high levels of circ-ANAPC7 (16). Yi et al. examined the expression of circ-VIM (circRNA derived from Vitementin) in bone marrow mononuclear cells (BMNCs) derived from the bone marrow of 113 AML patients and 42 healthy donors and observed upregulation of circ-VIM in AML patients (17). Moreover, they found that overexpression of circ-VIM was an independent poor prognostic factor and was markedly associated with shorter overall survival in AML patients. Ping et al. recently profiled the expression of circRNAs in the bone marrow of three AML patients and three controls (iron deficiency anemia) via circRNA-microarray analysis and documented elevated expression of circ_0009910 (hsa_circRNA_100053, circBase). circ_009910-miR-20a-5p upregulation was found in AML bone marrow patients compared to the control, and this upregulation predicted a poor outcome for AML patients. Further, the silencing of this circRNA inhibited the proliferation of AML cells and initiated apoptosis, highlighting the diagnostic and therapeutic potential of circ_009910-miR-20a-5p AML (18). Hsa_circ_0004277 (present on chromosome 10: 1125950-1126416) is encoded by WDR37, and the WDR37 family is involved in cellular events such as the cell cycle, apoptosis, and signaling pathways (3). Hsa_circ_0004277 was found to have reduced expression in both newly diagnosed untreated and relapsed AML patients (19). Interestingly, expression of hsa_circ_0004277 and WDR37 was found to be positively correlated during different AML stages, implicating WDR37 as a positive regulator of hsa_circ_0004277. Taken together, these reports suggest that circRNAs might be used as potential diagnostic and prognostic markers in AML.

**Table 1:** List of circular RNAs and their association with their respective miRNAs reported in AML.

**Name of circRNA**	**Circ base ID**	**Expression regulation**	**miRNA target sponge**	**Functions**	**Host gene**	**Target gene(s)**	**References**
Circ-ANAPC7	hsa_circRNA_101141	Up	miR-181 family	Oncogene, biomarker	ANAPC7	Numerous	(16)
circ-DLEU2	hsa_circ_0000488	Up	miR-496	Biomarker, Therapeutic target	DLEU2	PRKACB	(32)
–	hsa_circ_0004277-	Down	miR-138-5p miR-30c-1-3p miR-892b	Biomarker, Therapeutic target	WDR37 family	SH3GL2 PPARGC1A	(19)
circ-PAN3	hsa_circ_0100181	Up	miR-153-5p miR-183-5p	Drug resistance	PAN3	XIAP	(23)
circNPM1	hsa_circ_0075001	Up	miR-181	Biomarker	NPM1	TLR signaling pathway genes	(34)
–	hsa_circ_100290	Up	miR-203	Oncogene	SLC30A7	Rab10	(35)
circ_0009910	hsa_circRNA_100053	Up	miR-20a-5p	Biomarker	MFN2	RUNX3Rab27B Smad4	(18)

### circRNAs in Drug Resistance

One of the hallmarks of AML relapse following transient remission upon conventional treatment is the development of chemo-resistance to therapy, resulting in insensitivity to further treatment (20, 21). Multiple genes and non-coding sequences are involved in the development of drug resistance in AML (22). Various non-coding RNAs are emerging as important players in mediating drug resistance, and their targeting offers avenues for the development of novel treatment options. Nonetheless, studies on the potential involvement of deregulated circRNAs in AML drug resistance are just emerging. For example, circRNA-microarray expression analysis of doxorubicin (ADM)-resistant THP1 cells revealed a set of 49 circRNAs with altered expression, and elevated circPAN3 (product PAN3 gene) in recurrent and refractory AML was defined as a key player in drug resistance in AML (23). Of interest, circPAN3 is predicted to interact with **ten** miRNAs, miR-153-5p, miR-183-5p, miR-338-3p, miR-346, miR-545-3p, miR-574-5p, miR-599, miR-653-5p, miR-766-3p, and miR-767-3p. Functional analysis via the KEGG and GO databases predicted that the target genes of these miRNAs are associated with various cellular processes and signaling pathways involved in cancer (24–27). Moreover, miR-153-5p and miR-183-5p were found to interact with X-linked inhibitor of apoptosis protein (XIAP), which has been demonstrated as a drug resistance gene in AML (28, 29). Consistent with the predicted circPAN3-miRNA interactions, inhibiting circPAN3 expression reduces the expression of XIAP, and this effect was reversed by miR-153-3p- or miR-183-5p-specific inhibitors. In view of circPAN3 interaction with miR-153-5p and miR-183-5p, which in turn bind with XIAP, it can be predicted that circPAN3 may contribute to doxorubicin resistance in THP1 cells (23).

Similarly, overexpression of circPVT1 (derived from Plasmacytoma Variant Translocation 1 or PVT1) has also been associated with resistance to vincristine in AML (30), and knockdown of f-circM9 (a fusion circRNA) showed increased sensitivity to anti-leukemic drugs (31). These findings suggest that circRNAs can potentially be manipulated to reverse drug resistance (Figure 2A).

### circRNAs in Regulating Cell Proliferation and Apoptosis

Based on the circRNA microarray expression dataset GSE94591, it was previously shown that circ-DLEU2 (hsa_circ_0000488) was highly expressed in AML samples (37). circ-DLEU2 is the splicing product of the DLEU2 gene, which is located on chromosomes (32). Further studies demonstrated that circ-DLEU2 negatively regulated miRNA-496, whose downstream target gene is PRKACB (Figure 2B). The protein encoded by PRKACB is the catalytic subunit of cyclic AMP-dependent protein kinase, which regulates various signaling processes such as proliferation and differentiation through cAMP. The expression of PRKACB was negatively regulated by miRNA-496, while circ-DLEU2 promoted the expression of PRKACB by sponging miRNA-496 (32). Consequently, increased expression of circ-DLEU2 facilitated the *in vitro* proliferation of leukemic cells, blocked apoptosis, and promoted the formation of AML tumor *in vivo*. On the other hand, Morens et al. reported the deletion of the DLEU2 locus and embedded miRNA cluster miR-15a/16-1 in adult leukemia but its transcriptional repression due to hypermethylation in young AML patients (38). The DLEU2 gene contains the locus for miR-15a/16-1, the expression of which is negatively regulated by the binding of BSAP (a transcription factor) to the DLEU2 promoter. Increasing the expression of miR-15a/16-1 by inhibiting BASP facilitates apoptosis and leukemic cell cycle arrest (39). These findings suggest that circ-DLEU2 is crucial for *in vitro* proliferation and tumor formation in AML and, although the DLEU2 gene is identified as a tumor-suppressor gene, it can also be implicated in leukemogenesis.

Recently, Fan et al. reported that hsa_circ_100290, upregulated in AML, possesses oncogenic potential by enhancing cellular proliferation, inhibiting cell-cycle arrest, and suppressing cell apoptosis (35). They found that hsa_circ_100290 binds to miR-293, the expression of which is downregulated not only in AML but also in other cancers (40, 41). In many cancer types, miR-293 has been found to suppress the proliferation, invasion, and migration of cancer cells. Moreover, the target protein of miR-293 is Rab10, a member of the RAS superfamily of small GTPases (42). Furthermore, they found that the expression of miR-293 was decreased while Rab10 was elevated in AML patients, and hsa-circ_100290 expression was negatively and positively correlated with miR-293 and Rab10, respectively.

PVT1, on 8q24, is a non-protein coding region that produces numerous ncRNAs (43). The mechanistic role of the PVT1 region and its non-coding transcripts in cancers remains elusive. Apart from the role of PVT1 lncRNA in stabilizing MYC (44), several groups have reported the role of PVT1 as an oncogene in AML (45). Moreover, the circRNAs generated from exon 2 of the PVT1 region, termed circPVT1, possess oncogenic roles in AML by enhancing proliferation. Knocking down f-circRNAs9 (a product MLL/AF9) resulted in apoptosis, while its expression inhibited the drug-induced apoptosis of leukemic cells both *in vitro* and *in vivo* (31). To further corroborate these results, K562 cells were transduced with f-circRNA9 and treated with the standard drugs cytarabine (Arac-C) and ATO. Cells expressing f-circRNA9 inhibited the apoptosis induced by ATO and Arac-C (compared to empty vector-transfected cells) (31). These findings support the potential value of therapeutically targeting oncogenic circRNAs in AML (Figure 2B).

### Fusion-circRNAs in Leukemogenic Properties

Leukemia is characterized by prevalent chromosomal translocation events (46). These chromosomal rearrangements often result in the formation of fusion genes, which may produce not only oncogenic chimeric proteins (47) but also circRNAs known as fusion circRNA (f-circRNA) (Figure 1G) (48). The fusion chimeric gene MLL/AF9 yields two fusion circRNA products, f-circM9_1 and f-circM9_2, which promote leukemogenesis by promoting cell proliferation (31, 49). Hypoxia-inducible protein kinase (HIPK)-2 is a protein that stabilizes PML oligomerization, a process that is important in nuclear body formation. The disruption of nuclear bodies by the fusion protein of PML and the retinoic acid receptor α (RARα) (PML-RARα) is often associated with APL and is thus implicated in leukemogenesis (50). In another study, the mutation in HIPK2 showed its impaired localization with AML1 transcription factor complex. This complex serves as a target for leukemia-associated chromosomal translocations. The mutant HIPK2 negatively regulated AML1- and P53-dependent transcription and thus possibly contributes to the pathogenesis of leukemia (51). PCR amplification and sequencing of the site flanking the PML-RARα junction revealed that the paradigmatic fusion gene PML-RARα also produced multiple isoforms of f-circRNA with oncogenic potential (31). In this scenario, the circ-HIPK2 produced from the host gene might serve as a transcription co-activator in the formation of nuclear bodies. Moreover, investigating the circular RNA transcript of the fusion PML-RARα in the development of AML would constitute interesting future research.

Nucleophosmin 1 (NPM1) encodes a multifunctional, highly conserved nuclear chaperone protein that is involved in ribosomal biogenesis, apoptosis, and cell proliferation (52). An insertion into the exon12 of NPM1 produces an abnormal protein that abnormally localizes in the cytoplasm and contributes to leukemia formation in 35% of AML cases (37). A study showed the differential expression of several linear isoforms of the NPM1 gene in a cohort of AML patients (53). Moreover, the dislocation and deletion and high expression of NPM1 also contribute to the AML phenotype (48, 54). Recently, RNA-seq analysis identified circNPM1 variant hsa_circ_0075001 as a signature for defining patient subgroups. Comparative gene expression profiling of high vs. low hsa_circ_0075001 revealed several genes with differential expression. Gene expression pathway analysis of low and high circNPM1 expression groups revealed a significant difference in the TLR gene expression groups, particularly TLR4, TLR5, TLR7, and TLR8 (34).

## Web-Based Tools for circRNA Research Avenues

Because circRNA has multiple functions in various cancers, circRNA research is regarded as a hotspot in modern research. Various databases have been developed by researchers, mostly utilizing the data obtained from next-generation sequencing technology to shed light on the regulatory network and functional prediction of circRNA in various biological processes. The features of the most applied web-based tools are summarized in Table 2. The characteristics of the commonly employed databases are as follows. ***circBase***, developed by Glazar et al., collects and integrates data related to circRNAs from public references and hosts information from various species such as model organisms. The database not only provides free access to the user to browse and download gene annotations but also allows the user to obtain evidence supporting the expression details of a particular circRNA (55). ***CircNet*** is a freely accessible database developed by Liu et al., which incorporates the transcriptomic datasets of novel and previously published circRNAs. The database provides tissue-specific circRNA expression profiling in terms of a heatmap. Furthermore, CircNet also depicts the regulatory network between circRNA, miRNA, and a particular gene (56). ***CircInteractome***, developed by Dawood et al., is a freely accessible tool that enables the user to find the relevant miRNA- and RNA-binding protein sites on a particular circRNA. This database also facilitates circRNA-related research by allowing the researcher to design primers for qRT-PCR- and siRNA-based gene silencing. Moreover, CircInteractome can reveal the protein-coding abilities of a particular circRNA by deciphering its interaction with translational regulation factors and confirmation of IRES in circRNA (57). ***starBase*** depicts the interaction networks of circRNA with miRNA and RBP from 108 CLP-seq datasets generated by 37 different research studies. In addition, the other features of starBase v2.0 include miRNA-pseudogene interaction networks and miRNA-lncRNA interaction and competing endogenous RNA (ceRNA) functional networks (59). ***deepBase*** assembles NGS data from previous studies and enables users to annotate and discover small and long non-coding RNA and circRNAs. It also hosts features for decoding the evolution, expression profiles, and functions of non-coding RNAs across 19 species. Moreover, a spectrum of circRNA and lncRNA evolutionary patterns is also revealed by deepBase (60). Despite several worthwhile state-of-the-art toolkits for the identification, characterization, expression, and functional analysis of circRNA, problems such as a lack of a gold standard and overlap in predictions still need to be addressed. To this end, further innovation based on currently available methods with perfect precision and sensitivity should be pursued to overcome these challenges.

**Table 2:** Most commonly used databases containing circRNA-related information.

**Name**	**Website**	**Features**	**References**
circBase	http://www.circbase.org/	A merged and unified dataset containing sequence information of circRNAs	(55)
CircNet	http://circnet.mbc.nctu.edu.tw/	Tissue-specific circRNA expression profiles, circRNA-miRNA gene regulatory networks. Data is derived from transcriptomic sequencing studies	(56)
CircInteractome	https://circinteractome.nia.nih.gov/	Interaction of circRNA with miRNA and protein (RBP), primer and siRNA design	(57)
Circ2Traits	http://gyanxetbeta.com/circdb/	Interaction of circRNA with miRNA and long non-coding RNAs associated with disease or any other trait.	(58)
starBase	http://starbase.sysu.edu.cn/	Identify putative circRNA-miRNA and circRNA-RBP interaction networks	(59)
deepBase	http://deepbase.sysu.edu.cn/	Enable the annotation of sRNA, lncRNA, and circRNA.	(60)
circRNADb	http://reprod.njmu.edu.cn/circrnadb/circRNADb.php	A cancer-specific database. Enables the annotation of circRNA with protein-coding ability.	(36)
CIRCpedia	http://picb.ac.cn/rnomics/circpedia	Aims to annotate alternative back-splicing and alternative splicing in circRNAs	(61)
CIRCexplorer	https://circexplorer2.readthedocs.io/en/latest//	Aims to annotate alternative back-splicing and alternative splicing in circRNAs	(61)
CSCD	gb.whu.edu.cn/CSCD	Expression profiling of circRNAs obtained from RNA-seq data in cancer and normal tissues	(62)

## Future Perspective

### Mechanism Exploration

One of the major functions of circRNA is sponging miRNA, thus regulating gene expression at an mRNA level. The dysregulation of miRNAs is implicated in the initiation and progression of leukemia (63, 64). miRNA sponging by circRNA can either transform into a leukemogenic property or its inhibition depending upon the type of miRNA inhibited by the circRNA. Numerous miRNAs with oncogenic potential have been reported in hematopoietic malignancies, e.g., miR22 causes the repression of the TET2 gene, thus causing defective differentiation of leukemic cells (65). We expect future research to explore circRNAs with binding ability to oncogenic miRNA(s). Such research will be challenging, since multiple miRNAs can regulate a particular gene. For instance, an extensive network of TET2-targeting miRNAs (≥30 miRNAs) has been documented to inhibit the expression of the TET2 gene (66). These findings pave the way for the possible development of therapeutic strategies based on circRNA targeting mechanisms to rescue tumor suppressor gene expression.

### circRNA-RBP Sponge

Mounting evidence is revealing the interaction of circRNA with RNA binding proteins (RBPs) and its potential functional aspects (67). These RBPs include Argonaute (AGO) (4, 68), RNA polymerase (RNAPII) (5), Muscleblind protein (MBL) (69), Quanking I (QKI) (10), and Elongation Initiation Factor (EIF4A3) (57). These RBPs regulate gene expression and are thus involved in vital cellular processes. CRISPR-Cas9-based screening of RBP in AML has revealed a network of physically upregulated interacting RBPs that are crucial in regulating the splicing of RNA and maintaining the leukemic condition. The depletion of RBM39 (as the main regulator of this network) results in altered splicing of the mRNAs essential for AML, thereby causing the mortality of AML cells (70). Recently, the interaction of hsa_circ_0004870 with RBM39 has been shown to be associated with enzalutamide resistance in castration-resistant prostate cancer (71). This finding highlights the importance of investigating circRNA interactions with RBM in AML. In addition, mutational profiling of leukemic patients has uncovered somatic genetic mutations in RBP that are associated with splicing, as extensively reviewed by (72). Very recently, the high expression circMYBL2, a product of the MYBL2 gene, was reported in AML patients within internal tandem duplication (ITD) mutations in the FLT3 (FLT3-ITD) gene. A positive regulation was observed between circMYBL2 and FLT3-ITD mutant kinase. In terms of mechanism, circMYBL2 was found to enhance FLT3-ITD mutant protein expression by facilitating the binding of PTBP1 to the mRNA of mutant FLT3 kinase. The knockdown of circMYBL2 not only inhibits the proliferation and supports the differentiation of AML cells both *in vitro* and *in vivo* but also compromises the cytoactivity of cells with the FLT3-ITD mutation against quizartinib (Figure 2C) (73). Circ-Foxo3 binds to cyclin-dependent kinase 2 (CDK2) and cyclin-dependent kinase inhibitor 21 (p21), forming the circfoxo3-p21-CDK2 complex, thus preventing the cell cycle progression by inhibiting CDK2 (74) (Figure 2D). In AML, CDK2 blocks myeloid differentiation (Figure 2E). The ubiquitin (KLHL6-E3 ubiquitin ligase)-mediated proteasome degradation of CDK2 is associated with the activation of AML cell differentiation (75). The deletion of FOXO-1/3/4 family members facilitates the maturation of myeloid cells, AML cell death, and a diminution of leukemia-initiating cell function, thus improving animal survival (76). In the scenario of circ-FOXO3-p21-CDK2 ternary complex formation, it will be worthwhile to investigate the affinity of circ-FOXO3 or any other relevant circRNAs with CDK2 or any key cell cycle-regulating proteins, thus ultimately controlling the differentiation of cells in AML (Figure 2D). Molecular insights into understanding how malignancies are produced due to perturbation in pathways downstream of these sites will help in the identification of drug target regions.

Generally, owing to their stable structure, circRNAs are present in the cytoplasm, but some circular isoforms (EIcircRNA) are also present in the nucleus. These circular isoforms interact with chromatin modifiers, thereby causing repression or activation of the gene (77, 78). EIcircRNAs such as circEIF3J and circPAIP2 are associated with RNA polymerase II and recruit U1 small nuclear ribonucleoprotein (snRNP), which promotes the transcription of genes (8) (Figure 2F). In addition, certain circRNAs positively regulate the expression of their parent gene, such as in the case of the down-regulation of the ciRNA known as ci-ankrd52, which reduces the expression of ankrd52 without affecting the surrounding genes (Figure 2F) (5). circRNA derived from the SEP3 gene regulated the expression of the linear transcript by binding to its cognate DNA. The linear counterpart of circRNA-SEP3 with the same sequence binds to the DNA with weak affinity. Thus, circRNA-DNA formation presumably results in transcriptional repression and the generation of a SEP3 linear transcript with exon skipping (79). In addition, promoter-associated RNA (pRNA) suppresses the expression of the rRNA genes by recruiting DNMT3b to the target site of TTF-I (a transcription factor) via complementarity with the rDNA promoter (Figure 2F) (80). Watanabe et al. found that long non-coding RNA near the MYC gene could facilitate the amplification of MYC by influencing the movement of the replication fork (Figure 2E) (81). Another lncRNA known as ANRASSF1, produced from the opposite strand of a tumor-suppressor gene RASSF1 gene, binds with the promoter region of the parental gene, thus forming an RNA/DNA hybrid, and recruits polycomb repressive complex 2 protein (PRC2) to regulate its expression (82, 83). Like other ncRNAs, the circRNA may possibly affect DNA replication by binding with the genomic DNA, thus forming a DNA-RNA triplex (80). These findings highlight that circular and linear non-coding RNA may have the same binding pattern with the DNA and manifest unique effects on gene expression and DNA replication.

## Anti-Oncogenic Potential of circRNA

Some circRNAs, for instance, circ-ZEB1.19, circZEB-1.17, circZEB1.5, and circZEB1.33, by trapping miRNA-200, are involved in the suppression of lung cancer progression (56). The downregulation of circular RNA produced from mitochondrial translocation optimization 1 homolog (MTO1), known as circMTO1, was reported in hepatocellular carcinoma tissue. circMOT1 decoys miR-9 to allow the expression of tumor suppressor gene p21 (84). The transfection of synthesized small circular single-stranded DNA-9 (CSSD-9), which mimics the circRNA, resulted in increased expression of various tumor suppressor genes such as LF17, CDH1, and LASS2 by absorbing miR-9. The artificially synthesized CSSD-9, containing multiple binding sites for miR-9, impaired lung tumor progression and metastasis (85).

circRNAs were initially considered non-coding RNA due to their disability to recruit ribosome (9, 86). However, recent studies have documented the presence of an open reading frame (ORF) driven by the ribosomal machinery (13, 14). The circular transcript of the SNF2 histone linker PHD RING helicase (SHPRH) gene encodes a fully functional protein, 17kDa, termed circ-SHPRH, which suppresses the tumor transformation of glioma cells. In terms of mechanism, the circ-SHPRH protects SHPRH-146aa from ubiquitin-mediated proteasomal degradation, and subsequently, SHPRH ubiquitinates the proliferating cell nuclear antigen (PCNA) as an E3 ligase, thereby inhibiting cell proliferation (87). circRNA expression profiling of AML cell lines has identified that circKHLH8 is associated with clinical outcome, as its overexpression enhanced overall survival, with a higher level of platelets and reduced blast percentage in peripheral blood. Moreover, the inhibition of circFBXW7 facilitated the proliferation of AML cells, indicating the tumor-suppressive function of circFBXW7 in AML (88). Based on these findings, it is speculated that circRNA can work as an anti-oncogenic to promote the expression of tumor-suppressor genes by the derepression of miRNAs or by recruiting the transcription factor to the promoter of tumor suppressor genes.

## Targeted Therapy

Of note, the circRNA-miRNA-mRNA axis is a widely accepted mechanism in the establishment of leukemia. Circularization-based approaches can be used as a treatment strategy. Generally, a circRNA contains multiple miRNA binding sites (MRE). Therefore, targeting the inhibition of circRNA expression rather than a single miRNA/gene offers treatment advantages. Inhibiting circRNA expression will promote the protective effect of the corresponding miRNA in suppressing oncogenes, for instance, XIAP (23), Rab10 (35), PRACKB (32), and TLR pathway-related genes (34). CRISPR-Cas9-based engineering is a robust tool for performing functional studies of circRNA in cancer (89). However, depleting the circRNAs without affecting the existing genes is a challenge. In order to achieve circRNA-specific knockdown and avoid the targeting of circRNA from its linear RNA, the guide sequence must be designed to target the back-splice junction (BSJ) site that is uniquely present in circRNA to avoid its binding with the linear transcript. The non-protein-coding property of circRNA makes it a tough choice for depletion by incorporating insertions/deletions (INDELs). A CRISPR-Cas-assisted homologous recombination approach may overcome the first challenge by replacing the circRNA gene with a marker gene (90). Moreover, strategies to avoid the off-target effects should be considered to circumvent undesirable mutations (91). Nevertheless, CRISPR-Cas9-mediated deletion of the cdr1 locus has been achieved by Piwecka et al. (92). Similarly, disruption of the formation of RNA pairing reduces the expression of circRNA, and, under certain conditions, the knock-out of a circRNA has been achieved, for example, circGCN1LI in human PA1 cells (93). In addition, the currently developed tool for RNA targeting, CRISPR-Cas13, represents a promising method for targeting a specific circRNA (94). Cas13, a principal enzyme of the type VI CRISPR-Cas system guided by a 20 to 30-nt-long CRISPR RNA (crRNA), could specifically bind to the BSJ site and thus, in principle, be able to distinguish between the linear transcript and circular RNA. CRISPR-Cas-based circRNA engineering approaches can be harnessed for high-throughput screening (89), and future research using well-characterized guide RNA (gRNA) libraries designed for circRNAs may facilitate global screening studies.

## Challenges

**Figure 3:**
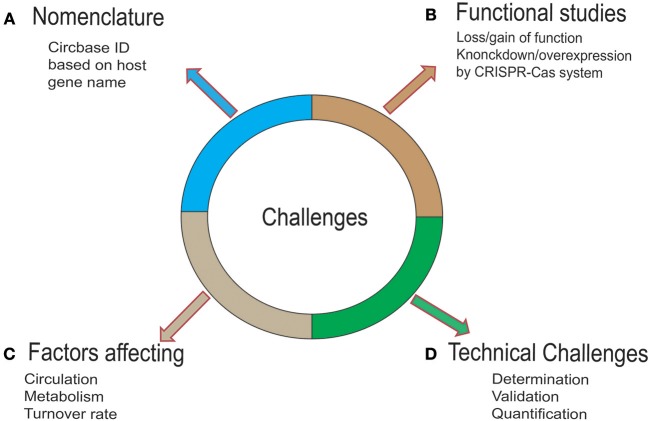
Challenges and strategies for circRNA. **(A)** To date, circRNAs have been named based on various parameters by various available databases, creating chaos in scientific research. Therefore, a standardized and uniform naming system is needed. **(B)** To gain mechanistic insights into the biological functions of circRNA, mechanism exploration is needed. To this end, an optimized CRISPR-Cas system would offer a robust and easy platform to carry out these studies. **(C)** The knowledge of the fate of circRNA in a cell is not fully known. Owing to the high stability of these molecules, the accumulation of circRNAs may exert toxic effects on the cell. Studies have shown that the surplus circRNAs are exported out of the cells in vesicles such as exosomes. Further molecular studies are needed to uncover the factors controlling the circulation, metabolism, and turnover of the circRNA. **(D)** Factors such as the peculiarity of the back-splice junction in circRNA, sequence similarity with the linear transcript, and low abundance compared to its linear counterpart make determination challenging. A multistep strategy for overcoming this challenge is promising. These steps include the discovery/identification of circRNA (by RNA-sequencing or microarray expression analysis), validation of circRNA by qRT-PCR, Sanger sequencing, Nothern Blotting, fluorescent *in-situ* hybridization (FISH), and the quantification of circRNA by targeted RT-qPCR assay.

Advancements in Next-Generation Sequencing (NGS) technologies and the availability of user-friendly bioinformatics tools have facilitated circRNA biology-related research in cancer. These advancements have helped in the transformation of circRNA research from considering it a redundant splicing byproduct to exploring its possible potential targeted therapeutic application in AML. However, the field is in its infancy and faces enormous challenges (Figure 3) and raises questions that need comprehensive consideration. First, the expense of NGS technologies limits the sample size, and there is a lack of gold-standard studies for data comparison. Second, despite extensive research related to the biogenesis of circRNA, knowledge on the metabolic processing of circRNA within cells is limited. Owing to the high stability of these molecules, the accumulation of circRNAs may have toxic effects on the cell. Studies have shown that surplus circRNAs are exported out of the cells in vesicles such as exosomes (95). Further molecular studies are needed to uncover the factors controlling the circulation, metabolism, and turnover of circRNA. Third, there is a lack of a gold-standard nomenclature system for circRNA. The naming of circRNA in future research should be based on the host gene along with the term “circ.” Fourth, lack of perfection in the web-based tools, characterized by the low correspondence of the circRNAs tested by these different tools, is not helpful. To this end, in the future, we expect the development of a database integrating more comprehensive information about function and molecular mechanism. Finally, our knowledge of circRNA in AML is limited. In the last couple of years, researchers have shown that the circRNA expression pattern is related of clinical outcome and a poor prognosis. The most common mode of action of circRNA in AML is miRNA sponging, underscoring the need for investigation of other biological functions of circRNA such as the circRNA-RBP and circRNA-DNA regulatory networks. Although current research is being focused on the validation of these findings, further studies should focus on questions such as how circRNAs contribute to AML and the correlation between altered expression of circRNA and the mutational analysis of candidate genes that are crucial in alternative splicing events.

## Conclusion

In conclusion, the role of circRNA in various cancers, including AML, is a major focus in cancer research. Although the detailed mechanisms of its biogenesis, transportation, function, and metabolism have not been fully studied, yet the glimpses provided by modern research suggest that circRNA as a potential candidate for AML diagnosis and therapy.

## Author Contributions

MJ, QZ, and LS designed and conceived the study. TS, BC, ZH, and TX helped with figure design and literature selection and drafted the manuscript. MF, LS, YW, and SP revised the language. QY helped in manuscript formatting and language revision. All of the authors read and approved the final manuscript.

### Conflict of Interest

The authors declare that the research was conducted in the absence of any commercial or financial relationships that could be construed as a potential conflict of interest.

## Abbreviations

AML: Acute myeloid leukemia
circRNA: Circular RNA
miRNA: Micro RNA
CeRNA: Competing endogenousRNA
f-circRNA: Fusion circRNA
EIcircRNA: Exonic-intronic circRNA
CiRNA: Circular intronic RNA
EcircRNA: Exonic circRNA
TLR: Toll-like receptor
PML-RARα: Promyelocytic leukemia and the retinoic acid receptor α (RARα)
IRES: Internal ribosome entry site
HIPK: Hypoxia-inducible protein kinase
XIAP: X-linked inhibitor of apoptosis protein
KEGG and GO: Kyoto encyclopedia of gene and genome and gene ontology
CLIP-seq: Crosslinking immunoprecipitation sequencing
qRT-PCR: Quantitative reverse transcriptase polymerase chain reaction
SiRNA: Small interfering RNA
CDK2: Cyclin-dependent kinase
AGO: Argonaute
QKI: Quanking I
EIF4A3: Elongation Initiation Factor
U1 snRNP: U1 small nuclear ribonucleoprotein
MRE, miRNA recognition element CRISPR-Cas9: Clustered regularly spaced palindromic repeats and CRISPR-associated gene/protein 9
NGS: Next-Generation Sequencing.
